# Biological Factors in the Workplace—Current Threats to Employees, the Effects of Infections, Prevention Options

**DOI:** 10.3390/ijerph19095592

**Published:** 2022-05-05

**Authors:** Anna Garus-Pakowska

**Affiliations:** Department of Nutrition and Epidemiology, Medical University of Lodz, 90-419 Lodz, Poland; anna.garus-pakowska@umed.lodz.pl

Infectious diseases or communicable diseases are spread from person to person by various routs. So we have airborne diseases, bloodborne diseases, and contact diseases. In the work environment, pathogens can spread from employee to employee, often leading to the development of an occupational disease [1].

According to the definition, an occupational disease is both acute and chronic disease, resulting from the performance of an occupation, resulting from the nature of the work or the conditions in which it takes place [2].

Biological agents in the workplace are classified according to their infectious effect. The criteria for this breakdown include:

- The possibility of causing disease in humans;
- The degree of risk to workers;
- Probability of spreading in the population;
- Possibility of prophylaxis and/or effective treatment [3].

Directive 2000/54/EC describes the principles of risk assessment, prevention, and control of biological agents at work. Biological agents are classified into four risk groups according to their level of risk of infection:

Group 1—Biological agents for which there is a low probability of causing disease in humans, and, therefore, they practically do not pose a threat to workers (e.g., weakened strains of bacteria used in the production of vaccines or yeasts intended for production purposes);

Group 2—Biological agents with documented harmful effects on the human body. They can pose a risk to workers but are unlikely to spread to the human population. In addition, there are methods of effective prevention or treatment. Examples include *Staphylococcus aureus* causing skin infections, *Borrelia burgdorferii* causing Lyme Disease and *Hepatitis A virus* (HAV);

Group 3—Biological agents that are dangerous to humans and can cause serious diseases. They pose a serious threat to workers and are very likely to spread through the population. The current state of knowledge enables the implementation of effective prevention and/or treatment of these factors. This group includes: *Mycobacterium tuberculosis*, *Hepatitis B virus* (HBV), *Hepatitis C virus* (HCV), *and Human immunodeficiency virus* (HIV). *SARS-CoV-2* was also included in group 3;

Group 4—Biological factors that cause severe disease in humans and most often lead to death. They pose a serious threat and their spread in the population of workers is very likely. At the same time, it is impossible to apply effective prophylaxis and treatment. This group includes only viruses: *Ebola* hemorrhagic fever virus, *Lassa* hemorrhagic fever virus and *Variola virus* [3].

Infectious diseases are an important part of the diseases to which workers are exposed.

In Poland, infectious diseases dominate among occupational diseases in recent years. Tuberculosis, HCV, and HBV are the most frequently diagnosed among healthcare workers. After all, not only medical personnel get sick. There are also teachers, officials, drivers, and all other employees who have contact with another person at the workplace (or optionally contact with an animal, e.g., foresters and farmers) [4].

The number of workers who become ill because they have had contact with the pathogen in the workplace is much greater. It is difficult to estimate how many because most of these events are unregistered. The ongoing COVID-19 pandemic has made this problem even worse.

In Poland, 2800 outbreaks of nosocomial infections were reported in 2020 (including 2265 outbreaks caused by SARS-CoV-2). Of the SARS-CoV-2 outbreaks, every fourth (25.5%, *n* = 577) outbreak related to staff reporting of infections. The number of healthcare workers infected with SARS-CoV-2 in connection with work in 2020 amounted to 20,697 people [5]. SARS-CoV-2 infections among healthcare workers have been and are reported worldwide [6,7,8,9]. Due to the way the virus is transmitted and its high contagiousness, there is no profession that is free from the risk of infection. However, private (non-professional) contacts cannot be ruled out in this case [10,11].

Another interesting example of an infectious occupational disease is Lyme disease. A tick bite can occur both in a farmer, a forester, and a paramedic who provided assistance to the injured in the accident. Lyme disease has been registered in many occupational groups, especially those working outdoors, including forestry workers, farmers, veterinarians, military recruits, and orienteers [12]. Currently, Lyme disease is the most frequently diagnosed occupational disease in the general population in Poland [4].

The specificity of infections varies depending on the profession. Among some employees, injuries with sharp tools are a significant problem, which may result in the transmission of blood-borne infection [13,14,15].

Postexposure prophylaxis (PEP) is recommended for workers who have an occupational exposure to blood, tissue, or other body fluids that may contain HCV, HBV, HIV, but also for workers exposed to tetanus or rabies [16,17,18].

Unfortunately, the knowledge of employees about the risk of infection, as well as methods and possibilities of infection prevention, is insufficient, which is emphasized by the authors in their publications [19,20].

Prophylaxis in the workplace may include non-specific methods, such as the use of personal protective equipment (gloves, masks), or washing and disinfecting hands, which is often emphasized as the most important method of interrupting the transmission of infectious agents [21]. There are also specific methods of preventing infections, and here we should mention vaccination of workers and methods of post-exposure prophylaxis. Manuscripts on this topic will also be very valuable for our Special Issue.

The new epidemic challenges are related to the psychological burden on employees. It is true that the stress of the global epidemic threat affects society as a whole. However, medical staff experienced higher levels of anxiety, depression, and insomnia [22]. These health problems affect hospital staff as well as primary care workers, where direct patient access has been limited in many countries due to the pandemic [23]. We invite authors who, in their manuscripts, will focus on the mental health of employees exposed to the risk of infection in the workplace.

Fighting infections in the workplace requires legal bases that control behavior, describe the obligations of employers, regulate the rules of prevention, and, finally, regulate conflicts between the employee and the employer (e.g., Directive 2000/54/EC [3] or Directive 2010/32/EU-prevention from sharp injuries in the hospital and healthcare sector [24]). Authors are also invited to submit manuscripts related to the legislation on infections.

To sum up, this Special Issue focuses on the prevalence of communicable diseases in the workplace. It can be divided into several thematic sections: (1) Occupational infections in the workplace (different occupational groups, different routes of spreading, various pathogens); (2) Prevention of infections in the workplace; (3) Psychological problems of employees related to the risk of infection in the workplace; (4) Legal aspects of infections in the workplace.

I have great pleasure to invite all authors to submit manuscripts for a Special Issue ”Infectious Diseases in the Workplace”. It will be a great compendium of the latest knowledge and developments in the field of workplace infection.
